# Gastrocnemius Myofiber Type and Mitochondrial Alterations Associated With Peripheral Artery Disease Severity

**DOI:** 10.1093/function/zqaf047

**Published:** 2025-10-06

**Authors:** Kate Kosmac, Rena Dana Wang, Jada Stewart, Parminder Kaur, Ahmed Ismaeel, Haseeb Ahsan, Lisa Hartnell, Esther E Dupont-Versteegden, Mary M McDermott, Robert L Sufit, Luigi Ferrucci, Charlotte A Peterson

**Affiliations:** College of Allied Health Sciences, Department of Physical Therapy, Augusta University, Augusta, GA, 30912, USA; Department of Biostatistics, College of Public Health, University of Kentucky, Lexington, KY, 40506, USA; Center for Muscle Biology, University of Kentucky, Lexington, KY, 40506, USA; College of Allied Health Sciences, Department of Physical Therapy, Augusta University, Augusta, GA, 30912, USA; Center for Muscle Biology, University of Kentucky, Lexington, KY, 40506, USA; Department of Physiology, College of Medicine, University of Kentucky, Lexington, KY, 40506, USA; College of Allied Health Sciences, Department of Physical Therapy, Augusta University, Augusta, GA, 30912, USA; National Institute on Aging, Baltimore, MD, 21224, USA; Center for Muscle Biology, University of Kentucky, Lexington, KY, 40506, USA; Department of Physiology, College of Medicine, University of Kentucky, Lexington, KY, 40506, USA; Department of Physical Therapy, College of Health Sciences, University of Kentucky, Lexington, KY, 40506, USA; Department of Medicine, Northwestern University Feinberg School of Medicine, Chicago, IL, 60611, USA; Department of Preventive Medicine, Northwestern University Feinberg School of Medicine, Chicago, IL, 60611, USA; Department of Neurology, Northwestern University Feinberg School of Medicine, Chicago, IL, 60611, USA; National Institute on Aging, Baltimore, MD, 21224, USA; Center for Muscle Biology, University of Kentucky, Lexington, KY, 40506, USA

**Keywords:** skeletal muscle, peripheral artery disease, myofiber type transition, mitochondria, ischemia, denervation

## Abstract

The extent of walking impairment varies among individuals with peripheral artery disease (PAD), which may reflect differences in the adaptability of lower extremity muscles to ischemia-reperfusion injury characteristic of the disease. Analyses of gastrocnemius muscle biopsies from 113 individuals with PAD [mean ankle-brachial index (ABI) = 0.65 ± 0.13, 38 (33.6%) women, 76 (67.2%) Black] showed a wide range of myofiber type distributions (9.6%-82.6% type 1 myofibers). The abundance of oxidative type 1 myofibers negatively correlated with ABI (*r* = −0.22, *P =* 0.02), a measure of PAD severity. The abundance of type 1 myofibers also negatively correlated to 2a/x myofiber abundance (*r* = −0.76, *P* < 0.001). Eighty % of participants had NCAM+ myofibers, a potential indicator of myofiber denervation. Overall, 3.2% of total myofibers were NCAM+. Of 113 muscle biopsies, 86 (76.1%) contained type 1 myofibers with regions lacking intermyofibrillar mitochondria (IMFM-), which may represent formation of target myofibers. In type 1 myofiber IMFM- areas, 77.8% contained 2x myosin heavy chain and/or the autophagy marker LC3. Electron microscopy within one muscle with IMFM- myofibers confirmed sarcomere disruption in IMFM- regions. These analyses support the possibility of type 2 myofibers transitioning to type 1 in PAD and suggest IMFM- target fibers may represent visualization of this process for the first time. Because type 1 myofibers are more resistant to oxidative damage, results suggest the possibility that a higher proportion of type 1 myofibers in PAD with increasing disease severity may be a compensatory mechanism to maintain muscle.

## Introduction

Peripheral artery disease (PAD) is defined by atherosclerotic arterial narrowing, limiting blood flow to the lower extremities.^1,2^ People with PAD have greater mobility impairment, lower physical activity, and a faster decline in walking ability than those without PAD.^3-8^ With PAD, lower extremity ischemia during walking and reperfusion at rest damages skeletal muscle and greater muscle damage is associated with higher rates of mobility loss.^9^

The distribution of myofiber types, defined by the myosin heavy chain (MyHC) isoform expressed, is associated with muscle strength, power, endurance, and fatiguability, alongside key factors such as muscle size, neural activation, and metabolic capacity. In human lower-limb muscles, like the gastrocnemius commonly studied in PAD, type 1 slow-oxidative myofibers predominate in endurance-oriented roles and exhibit high fatigue-resistance due to abundant mitochondria and capillaries. Type 2 fast myofibers include oxidative-glycolytic type 2a, with substantial mitochondrial content and considerable fatigue resistance, and the more fatigable glycolytic type 2x, infrequent in humans compared to rodents where extreme type 2b equivalents are common. This nuanced distribution, including hybrid fibers co-expressing MyHC isoforms, influences physical function^10^ and can adapt to stimuli such as exercise, aging, denervation, and disease.^11-14^ Myofiber transitions from type 1 to type 2 have been documented with hyperthyroidism, denervation, and chronic obstructive pulmonary disease (COPD).^14,15^ Type 2 to type 1 myofiber transitions have been reported with hypothyroidism, hibernation, and aging.^14,16^ In PAD, reports indicate selective type 2 myofiber atrophy and denervation, alongside relative preservation or elevation of oxidative type 1 myofibers.^17-19^ However, data on myofiber type distribution in PAD muscle remain conflicting, with some studies reporting lower type 2 myofibers,^18,19^ some reporting higher type 2 myofibers,^20-22^ and some reporting no difference compared to control.^23-25^ The present study elucidates this heterogeneity in myofiber type distribution, which may account for prior discrepancies in smaller cohorts, and identifies novel characteristics suggestive of adaptive remodeling, including potential MyHC transitions.

Heterogeneity in myofiber type distribution was reported in a small sample of individuals with PAD (*n* = 26), with the proportion of type 1 myofibers ranging from 9% to 81% in gastrocnemius muscle biopsies.^26^ A higher proportion of type 1 myofibers was associated with abnormal myofibers that lacked intermyofibrillar mitochondria (IMFM-), possibly representing target myofiber formation, which was not observed in muscle from individuals without PAD.^26^ Therefore, the hypothesis of the current study is that heterogeneity in myofiber type distribution represents interindividual differences in adaptive remodeling of myofiber type in response to PAD. Further, the appearance of IMFM- target myofibers may be related to this process, given the positive association with the proportion of type 1 myofibers.^26^

To explore myofiber type distribution and the presence of IMFM- myofibers in PAD, gastrocnemius muscle samples from 113 individuals with PAD were evaluated. We expected to observe heterogeneity in myofiber type distribution among individuals and hypothesized positive type 1 myofiber associations with disease severity assessed by ankle-brachial index (ABI). We note that ABI is a validated diagnostic tool in PAD^27,28^ but does not capture microvascular perfusion deficits that may independently affect skeletal muscle oxygenation and myofiber type distribution. In this study, we use ABI to stratify individuals with PAD by disease severity. Understanding differences in myofiber type distribution and phenotype may provide insight regarding muscle adaptations to progressing PAD.

## Materials and Methods

### Registration and Participant Information

Baseline muscle biopsies from 10 observational or randomized PAD trials at Northwestern University were analyzed for this study (https://clinicaltrials.gov/): PROPEL (NCT01408901),^29^ COCOA-PAD pilot (NCT02876887),^30^ LITE (NCT02538900),^31^ TELEX (NCT02593110),^32^ WALCS III (NCT00520312),^33^ BRAVO (NCT02276781),^34^ GOALS (NCT00693940),^35^ RESTORE (NCT02246660),^36^ PERMET (NCT03054519), and HI-PAD (NCT03363165).^37^ All studies were approved by the institutional review board at Northwestern University and carried out according to the Declaration of Helsinki. All participants provided written informed consent. Detailed inclusion and exclusion criteria for each study can be found in the published clinical trial results.^29-37^ PAD was defined as a baseline ABI < 0.90, ABI ≥ 0.90 and ≤ 1.00 with ≥ 20% ABI drop after a heel rise test, or ABI ≥ 0.91 but vascular lab or angiographic evidence of PAD (Table S1).^38,39^ In this study, we use ABI to stratify disease severity and acknowledge ABI is not a direct proxy measure for ischemic burden within the gastrocnemius muscle tissue.

For electron microscopy, a gastrocnemius biopsy was obtained from a healthy volunteer, aged 67, at the National Institute on Aging following the Tissue Procurement protocol #03AGN322.

### Muscle Biopsies, Immunohistochemistry, and Histochemistry

Open muscle biopsies were obtained from the medial head of the gastrocnemius, as described in detail elsewhere.^26^ Approximately 100 mg of tissue was embedded in tragacanth gum on cork, frozen in liquid-nitrogen cooled isopentane and stored at −80°C. Muscle samples are labeled with an arbitrary ID and shipped on dry ice, followed by immediate storage back at −80°C. Biopsies were sectioned in a cryostat with a chamber temperature of −22 to −25°C at a thickness of 7 µm and sections were placed onto SuperFrost Plus slides (Thermo Fisher Scientific, Waltham, MA #12-550-15). Prior to immunohistochemical or histochemical analyses, slides were removed from storage at −20°C and air dried at room temperature for at least 10-15 min.

#### Myofiber Type Distribution and Minimum Feret Diameter

Myofiber type distribution was determined via our previously published and validated protocol.^40^ Primary antibodies against MyHC isoforms (Developmental Studies Hybridoma Bank (DSHB) (Iowa City, IA, USA) included mouse IgG2B BA.D5 concentrate (Type 1, 1:100, #BA.D5-c, RRID: AB_2235587), mouse IgG1 SC.71 supernatant (Type 2a, 1:50, #SC.71-s, RRID: AB_2147165), and mouse IgM 6H.1 supernatant (Type 2x, neat, #6H.1-s, RRID: AB_1157897). Myofiber borders were delineated using rabbit anti-laminin (1:100, MilliporeSigma, Burlington, MA, USA #L9393, RRID: AB_477163). Secondary antibodies from Thermo Fisher were used for visualization of myosin isoforms and included: anti-mouse IgG2b Alexa Fluor 647 (1:250, #A21242, RRID: AB_2535811), IgG1 Alexa Fluor 488 (1:250, #A21121, RRID: AB_2535764), and IgM Alexa Fluor 555 (1:250, #A21426, RRID: AB_2535847). Laminin labeling of myofiber borders was amplified with Biotin-SP (long spacer) AffiniPure Goat Anti-Rabbit IgG (H + L) (min X Hu, Ms, Rat Sr Prot) (1:1000, Jackson ImmunoResearch Labs Cat# 111-065-144, RRID:AB_2337965) followed by Streptavidin-AMCA (1:150, Vector Laboratories Cat# SA-5008, RRID:AB_2336103). Myofiber type-specific minimum feret diameter (MFD) and myofiber type distribution were processed and analyzed with MyoVision automated software.^41^ The number of total fibers quantified across samples ranged from 142 to 4120 with a mean of 631.

#### Identification and Characterization of Myofibers Devoid of Intermyofibrillar Mitochondria (IMFM- Target Myofibers)

To identify IMFM- myofibers, cryosections were dried for 10 min post-sectioning followed by overnight incubation in succinate dehydrogenase (SDH) solution (nitrotetrazoleium blue (Sigma, N6876), succinic acid disodium salt (Sigma, 224731), 0.2 M phosphate buffered saline) at 37°C in a shaking water bath, according to published, validated methods.^42,43^ Following incubation, slides were dipped for 1 min in 30%, 60%, and 30% acetone then washed with 10 mM PBS, pH7.4. Slides were blocked in 2.5% normal horse serum with 0.5% Tween-20 for 1 h then incubated overnight in a cocktail containing the type 1 and 2x MyHC antibodies listed above, as well as anti-rabbit LC3B (1:100, Novus Cat# NB100-2220, RRID:AB_10003146).

Total myofibers and myofibers expressing either type 1 or type 2x MyHC were manually quantified for each sample; type 2a myofiber numbers were determined by subtracting the sum of type 1 and 2x myofibers from the total. IMFM- target myofibers were quantified using the following parameters: (1) IMFM- areas must have clear boundaries identifying a distinct area devoid of SDH activity; areas appearing “moth eaten” or where SDH is present at low intensity were not counted as IMFM-; (2) areas lacking SDH activity must be greater than 40 µm in diameter to be counted as IMFM-; and (3) IMFM- areas must be confirmed by two independent, experienced researchers. IMFM- target myofibers were quantified by myofiber type and expressed as a percentage of total myofibers, type 1 myofibers, or type 2 (2a and 2ax) myofibers. IMFM- areas were characterized for the expression of 2x MyHC alone (2x+), LC3 alone (LC3+), both 2x MyHC and LC3 (2x + LC3+), or neither 2x MyHC nor LC3 (2x-LC3-). Data were counted as individual events and expressed as a percentage of type 1 IMFM- target myofibers or type 2 IMFM- target myofibers. IMFM- target myofiber counts were performed on 3-4 sections per sample and averaged across serial sections. The number of total myofibers quantified per sample ranged from 185 to 3614 with a mean of 656.

#### Visualization of Mitochondrial Complex IV and Complex I in IMFM- Areas

Immunohistochemistry for mitochondrial complexes was performed following fixation of sections for 10 min with 4% paraformaldehyde (PFA). Sections were then washed in 50 mM tris buffered saline (TBS) and blocked for 1 hour in TBS containing 1% normal goat serum and 1% Tween-20. Following blocking, slides were washed in TBS then incubated with the following primary antibodies in TBS, 5% NGS, 0.5% Tween-20 overnigh without rocking: MTCO1 [1D6E1A8] (1:50, Abcam Cat# ab14705, RRID:AB_2084810) and NDUFB8 [20E9DH10C12] (1:100, Abcam Cat# ab110242, RRID:AB_10859122). Slides were then washed with TBS and incubated for 1 h with anti-mouse IgG2a Alexa Fluor 488 (1:250, Thermo Fisher Scientific Cat# A-21131, RRID:AB_2535771). NDUFB8 was amplified by incubation for 1 h in Biotin-SP-AffiniPure Goat Anti-Mouse IgG, Fc_Subclass 1 Specific (1:1000, Jackson ImmunoResearch Labs Cat# 115-065-205, RRID:AB_2338571) followed by 1 h in Streptavidin-horse radish peroxidase (SA-HRP) (1:500 of 2.5 µg/µl stock, Thermo Fisher, #S-911) then 20 min in SuperBoost TSA AlexaFlour 594 (1:500, Thermo Fisher, #B40957).

#### Myofiber Type-Specific Expression of Neural Cell Adhesion Molecule

Neural cell adhesion molecule was used as a preliminary indicator of denervation, acknowledging the potential non-specificity and complementary morphological assessemetns employed in other studies.^44,45^ NCAM+ myofibers were identified by incubating cryosections with primary and secondary antibodies used above to identify type 2a and 2x expressing myofibers (listed above), in addition to anti-rabbit NCAM (1:50, Millipore Cat# AB5032, RRID:AB_11213653) followed by goat anti-rabbit Alexa Fluor 488 (1:200, Thermo Fisher Scientific Cat# A-11034, RRID:AB_2576217). Total myofibers, myofibers expressing both 2a and 2x MyHC (2ax), and myofibers expressing 2a MyHC only were quantified; myofibers expressing neither 2a nor 2x MyHC were counted as type 1 myofibers. The number of type 1, 2a, or 2a/x myofibers positive for NCAM were quantified and expressed as a percentage of each myofiber type. The number of total myofibers quantified across samples ranged from 140 to 3770, with a mean of 792.

For a detailed list of all antibodies used for immunohistochemistry, see the Major Resources Table in the Supplemental Material. For all immunohistochemistry and histochemistry, stitched images of entire biopsy cross-sections were acquired with a 20x objective using either an Axio Imager M2 (Carl Zeiss, Oberkochen, Germany) equipped with ZEN software (blue edition, v2.3) or an Olympus BX61VS (Olympus, Tokyo, Japan).

### Transmission Electron Microscopy

Gastrocnemius muscle was weighed then trimmed for orientation, fixed in 2% glutaraldehyde, 3% PFA in sodium cacodylate buffer, pH 7.4 for 1 h, then rinsed 3 × 5 min in sodium cacodylate buffer. Samples were post-fixed with 2% osmium tetroxide in sodium cacodylate buffer reduced with potassium ferrocyanide (3% w/v) for 1 h, then washed with sodium cacodylate buffer 3 × 5 min, and rinsed again in double distilled water. Samples were en bloc stained using 2% uranyl acetate in water overnight. The tissue was dehydrated in a graded series of ethanol from 30 to 100% then embedded in epoxy resin and cured at 60°C. Sections between 70-80 nm in thickness were cut from the cured blocks using a Leica UC7 ultramicrotome (Leica Microsystems, Deerfield IL). Sections were placed on formvar-carbon coated nickel grids, stained with 5% uranyl acetate and lead citrate and images acquired on a T12or L120C transmission electron microscope using 120 kV (FEI, Holland, The Netherlands).

### Statistical Analyses

All myofiber-level data was aggregated into a single value for each subject; therefore, no hierarchical structure was present within the data. Continuous variables were summarized with mean and standard deviation (SD). Normality was determined using D’Agostino & Pearson and Shapiro-Wilk tests. Spearman Rho correlation coefficients (r) were estimated to quantify relationships among muscle characteristics. The 95% confidence intervals (CI) for correlation coefficients were determined using Fisher’s Z transformation. Due to the exploratory nature of our analysis, corrections for multiple testing were not applied. Statistical analysis between two measures was determined using the Wilcoxon signed-rank test. One-way analysis of variance (ANOVA) using Friedman test with post hoc tests was used to assess myofiber type-specific size. All muscle features analyzed represent a single subject-level value. Statistical analyses were performed using R version 4.4.2 and verified using SAS, graphs were made using Prism 10.3.1 (GraphPad Software Inc, La Jolla, CA, USA). All tests were two-tailed, with significance set at an α < 0.05.

## Results

The demographic and clinical characteristics of the study population are shown in Table 1. Participants had a mean age of 68.05 ± 7.97 years and a mean ABI of 0.65 ± 0.13; 33.6% were women, 67.2% were Black, and 30.1% were White. In this cohort of participants, 35.4% had diabetes, 50.4% were current smokers, and 22.1% had intermittent claudication.

**Table 1.** Physical and Clinical Characteristics of PAD Participants

PAD (*n* = 113)	Mean ± SD	Range
Mean age (years)	68.0 ± 8.0	51.4-87.8
ABI	0.65 ± 0.13	0.36-0.94
BMI (kg/m^2^)	28.6 ± 5.9	18.3-44.0
Sex (women, %)	33.6	
Race (%)		
Black	67.2	
White	30.1	
Other	2.7	
Diabetes (%)	35.4	
Hypertension (%)	81.4	
Chronic heart failure (%)	6.2	
Statin use (%)	68.1	
Current Smoker (%)	50.4	
Intermittent claudication (%)	22.1	

Values are mean ± SD; ABI = ankle-brachial index; BMI = body mass index.

### Heterogeneity in Myofiber Type Distribution Across 113 Muscles From Individuals With PAD and Association With Disease Severity

**Figure 1.**
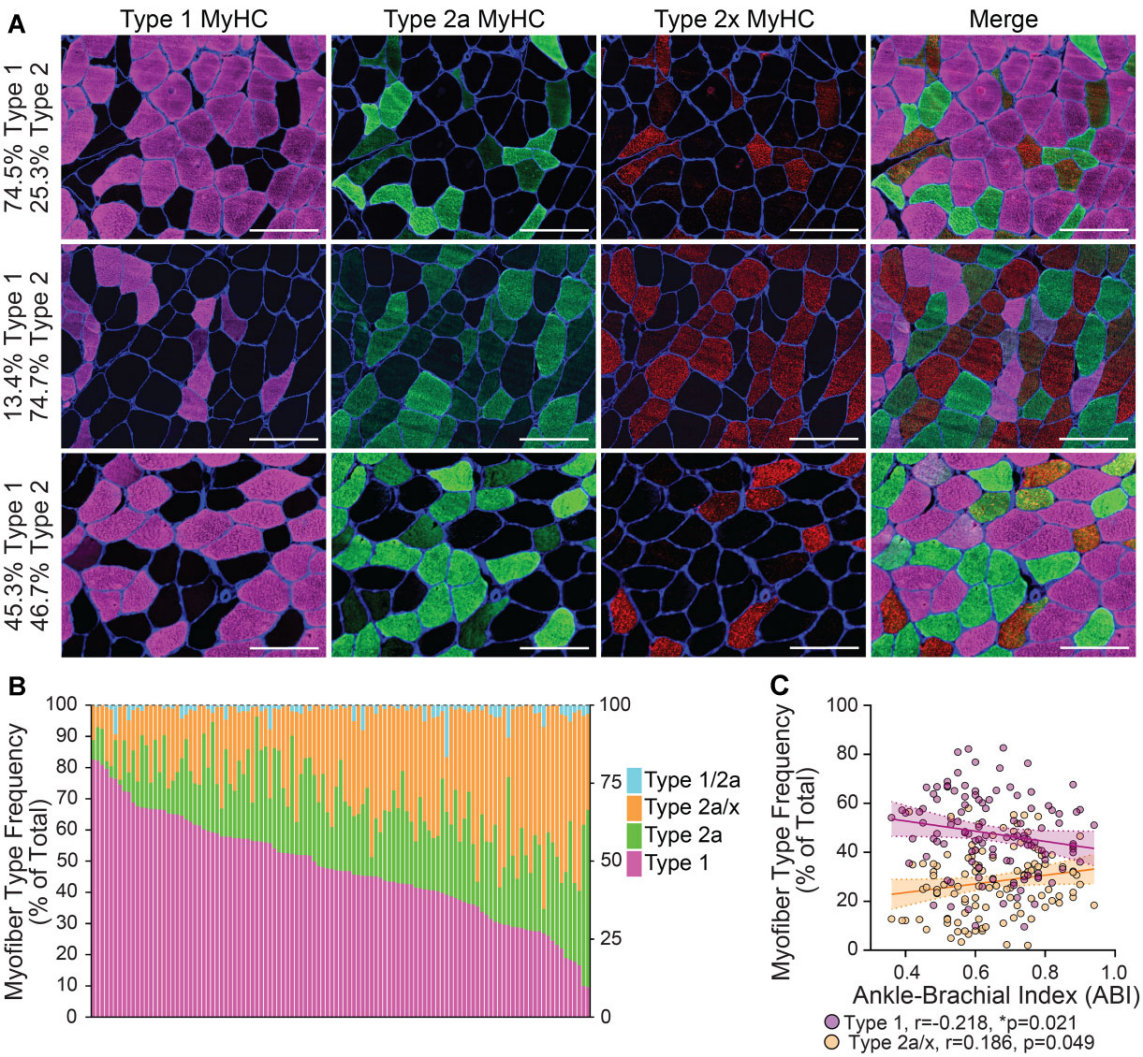
Heterogeneous gastrocnemius myofiber type across individuals with PAD. A) Representative regions of interest (ROIs) showing the expression of Type 1, Type 2a, Type 2x myosin heavy chain (MyHC) in gastrocnemius muscle from individuals with PAD; myofiber borders/Laminin. The percentage of myofiber types within the biopsy was quantified and is shown on the left; top: mostly Type 1, middle: mostly Type 2 (2a & 2a/x), bottom: evenly distributed Type 1 & Type 2. ROIs taken from images acquired at 200x, scale bar = 100 µm. B) Bar graph quantification showing myofiber type distribution (percentage of total myofibers quantified) across 113 individuals with PAD. Colored bars represent the percentage of myofibers expressing Type 1, Type 2a, hybrid Type 2a/x, and hybrid Type 1/2a MyHC within each muscle sample. C) Representative dot plot and linear regression lines showing associations between PAD severity, measured by ankle-brachial index (ABI), and the percentage of myofibers expressing either Type 1 or hybrid Type 2a/x MyHC across 113 individuals with PAD. Associations determined by Spearman Rho, r = correlation coefficient, error bands represent the 95% confidence interval for regression.

The distribution of type 1, type 2a, and type 2a/x myofibers was heterogeneous, with large interindividual differences across PAD muscles. Of the 113 muscles, 23.9% of individuals had > 60% type 1 myofibers (Figure 1A, top), 30.1% of participants had > 60% type 2a and 2a/x myofibers (Figure 1A, middle), and 46.0% had approximately 50% type 1 and 50% type 2a and 2a/x myofibers (Figure 1A, bottom). The myofiber type distribution, summarized in the bar graph of Figure 1B shows interindividual differences across muscles from people with PAD. Among all 113 participants, the proportion of total myofibers that were type 1 ranged from 9.6% to 82.6%, type 2a ranged from 1.6% to 56.9%, and type 2a/x ranged from 1.9% to 58.3%. Myofibers expressing type 1 and 2a MyHC (1/2a) comprised 2.1% of total myofibers on average and ranged from 0.0% to 16.6% (Figure 1B). Myofibers expressing only 2x MyHC were not detected. More severe PAD (ie, lower ABI values) was associated with higher proportions of type 1 myofibers (*r* = −0.22, *P* = 0.02) (Figure 1C). However, this association did not remain statistically significant when adjusting for smoking status (*r* = −0.16, *P* = 0.11, *n* = 102). Conversely, less severe disease (higher ABI) was associated with higher proportions of type 2a/x myofibers (*r* = 0.19, *P* = 0.05) (Figure 1C). These findings suggest myofiber types may shift with increasing severity of PAD and smoking may contribute to myofiber shifts, with higher proportions of type 1 myofibers associated with lower ABI and current smoking status.

Measurement of myofiber size using MFD revealed type 1 myofibers averaged 73.33 µm, type 2a/x myofibers averaged 68.91 µm, and type 2a myofibers averaged 65.08 µm (Figure S1A). One-way analysis of variance (ANOVA) determined that type 1 myofibers were significantly larger than either type 2a myofibers (adj. *p* = 0.001) or type 2a/x myofibers (adj. *P* = 0.002). The mean MFD (including all myofiber types) was positively associated with the proportion of type 1 myofibers (*r* = 0.26, *P* = 0.005) and negatively associated with the proportion of type 2a myofibers (*r* = −0.19, *P* = 0.04) (Figure S1B).

### Myofibers Lacking Intermyofibrillar Mitochondria (IMFM- target myofibers) Were Common in PAD Muscle and Are Predominantly Type 1

**Figure 2.**
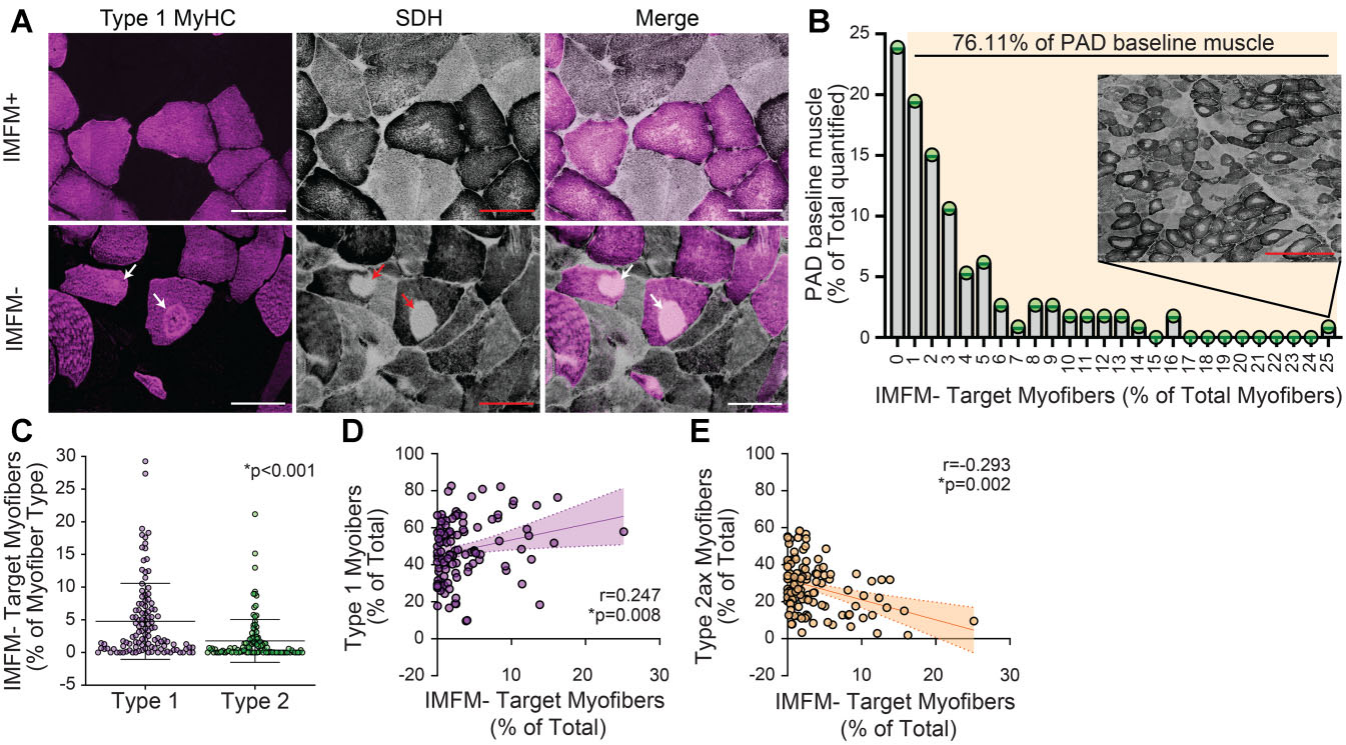
Presence of target myofibers devoid of intermyofibrillar mitochondria (IMFM-) in gastrocnemius muscle from PAD patients. A) Representative ROIs showing Type 1 MyHC and mitochondrial activity, delineated using succinate dehydrogenase (SDH), in gastrocnemius muscle from PAD individuals. Notice target myofibers devoid of intermyofibrillar mitochondrial activity (IMFM-) in the bottom panel (white or red arrows). ROIs taken from images acquired at 200x, scale bar = 200 µm. B) Histogram depicting the proportion of PAD muscle samples displaying IMFM- target myofibers (quantified as the percentage of total myofibers counted). Approximately 76% of all samples analyzed displayed some proportion of IMFM- target myofibers, *n* = 113. Inset: Representative ROI showing a PAD patient with a high proportion of IMFM- target myofibers (25% of total fibers); scale bar = 1 mm. C) Dot plot quantification of IMFM- target myofibers by myofiber type across 113 PAD gastrocnemius muscle samples showing more Type 1 myofibers are IMFM- target myofibers. Data expressed as mean ± SD, *P*-values determined using Wilcoxon signed-rank test. D-E) Associations of IMFM- target myofibers with D) the percentage of Type 1 and E) the percentage of 2a/x myofibers. Note as Type 1 myofibers increase, IMFM- target myofibers increase while as Type 2a/x myofibers increase, IMFM- target myofibers decrease. Associations determined using Spearman Rho, *r* = correlation coefficient, *n* = 113; error bars represent the 95% confidence interval of the linear regression.

Of the 113 muscle specimens, 102 contained myofibers with IMFM- areas, identified by the absence of succinate dehydrogenase (SDH) activity, similar to descriptions of target myofibers in other studies (Figure 2A).^46-48^ Approximately, three fourths (76.1%) of PAD muscles displayed ≥1% of myofibers with areas devoid of intermyofibrillar mitochondria (IMFM- target myofibers) (Figure 2B). The prevalence of IMFM- target myofibers varied across individuals. While 66.4% had IMFM- areas in <3% of total myofibers, 10.6% of samples contained >10% of total myofibers with IMFM- areas. In one participant, IMFM- target myofibers comprised 25.2% of myofibers (Figure 2B, inset). Type 1 IMFM- target myofibers comprised 64.5% of total IMFM- target myofibers and the proportion of type 1 myofibers with IMFM- areas was significantly greater than type 2 myofibers (4.8% of type 1 myofibers versus 1.8% of type 2 myofibers, *P* < 0.001) (Figure 2C), consistent with prior studies reporting target myofibers are predominantly type 1.^49^ Larger myofiber size, for all myofiber types, was positively associated with the abundance of IMFM- target myofibers (mean: *r* = 0.24, *P* = 0.011; type 1: *r* = 0.22, *P* = 0.017; type 2a: *r* = 0.21, *P* = 0.035; type 2a/x: r = 0.29, *P* = 0.003) (Figure S1B). A higher proportion of total IMFM- target myofibers was associated with a higher proportion of type 1 myofibers (*r* = 0.25, *P* = 0.008) and a lower proportion of type 2a/x myofibers (*r* = −29, *P* = 0.002) (Figure 2D-E). Together, these results demonstrate that IMFM- target myofibers are associated with larger myofiber size and are more common in type 1 myofibers, consistent with previously published findings.

### Altered Mitochondrial Complex Abundance in IMFM- Areas and Ultrastructural Alterations Within Muscle From PAD Individuals

**Figure 3.**
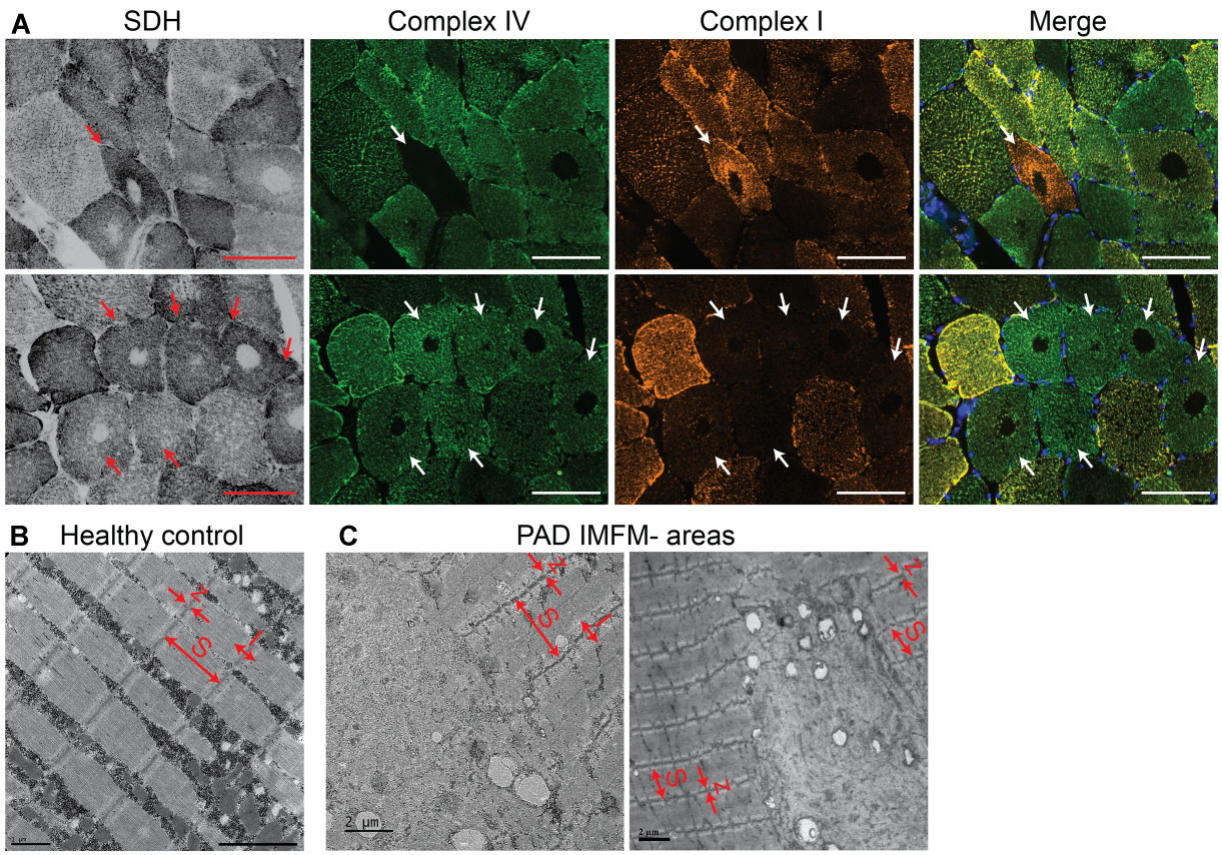
Absence of mitochondria in IMFM- areas and disorganized sarcomere organization PAD gastrocnemius muscle. A) Representative ROIs depicting mitochondrial activity (SDH) and mitochondrial complex IV and complex I subunit proteins in PAD gastrocnemius muscle. IMFM- areas within target myofibers lack mitochondrial complex subunit proteins. Additionally, several IMFM- target myofibers express only one of the two mitochondrial complex subunits (arrows). ROIs acquired from images taken at 200x, scale bars = 100 µm. B-C) Representative transmission electron microscopy (TEM) showing normal sarcomere organization in B) healthy control muscle and C) sarcomere disorganization consistent with IMFM- target myofibers in muscle from an individual with PAD. S = sarcomere, z = z-line, and I = I-band; scale bars: healthy control = 1 µm, PAD = 2 µm.

SDH is a component of mitochondrial complex II. To determine whether IMFM- areas resulted from loss of complex activity or loss of the entire mitochondrion, complex IV (cytochrome c oxidase) and complex I membrane subunit proteins were assessed. Within IMFM- regions, there was no detectable complex IV or complex I (Figure 3A), suggesting a complete absence of mitochondria. Myofibers expressing either complex IV or complex I, but not both, were also present (Figure 3A, white arrows). Transmission electron microscopy (TEM) was used to visualize the ultrastructural organization of the sarcomere within IMFM- areas. Muscle from a healthy, older adult showed normal sarcomere (S) organization with distinguishable I-bands (I) and Z-lines (z) (Figure 3B). In a PAD muscle containing 3.9% IMFM- myofibers, sarcomere organization was absent and the myofibers lacked mitochondria (Figure 3C), suggesting that IMFM- areas both lack mitochondria and contain disrupted sarcomeres.

### Inclusion of 2x MyHC And/Or the Autophagy Marker LC3 Within Type 1 IMFM- target Myofibers and Disruption of Type 1 Myosin

**Figure 4.**
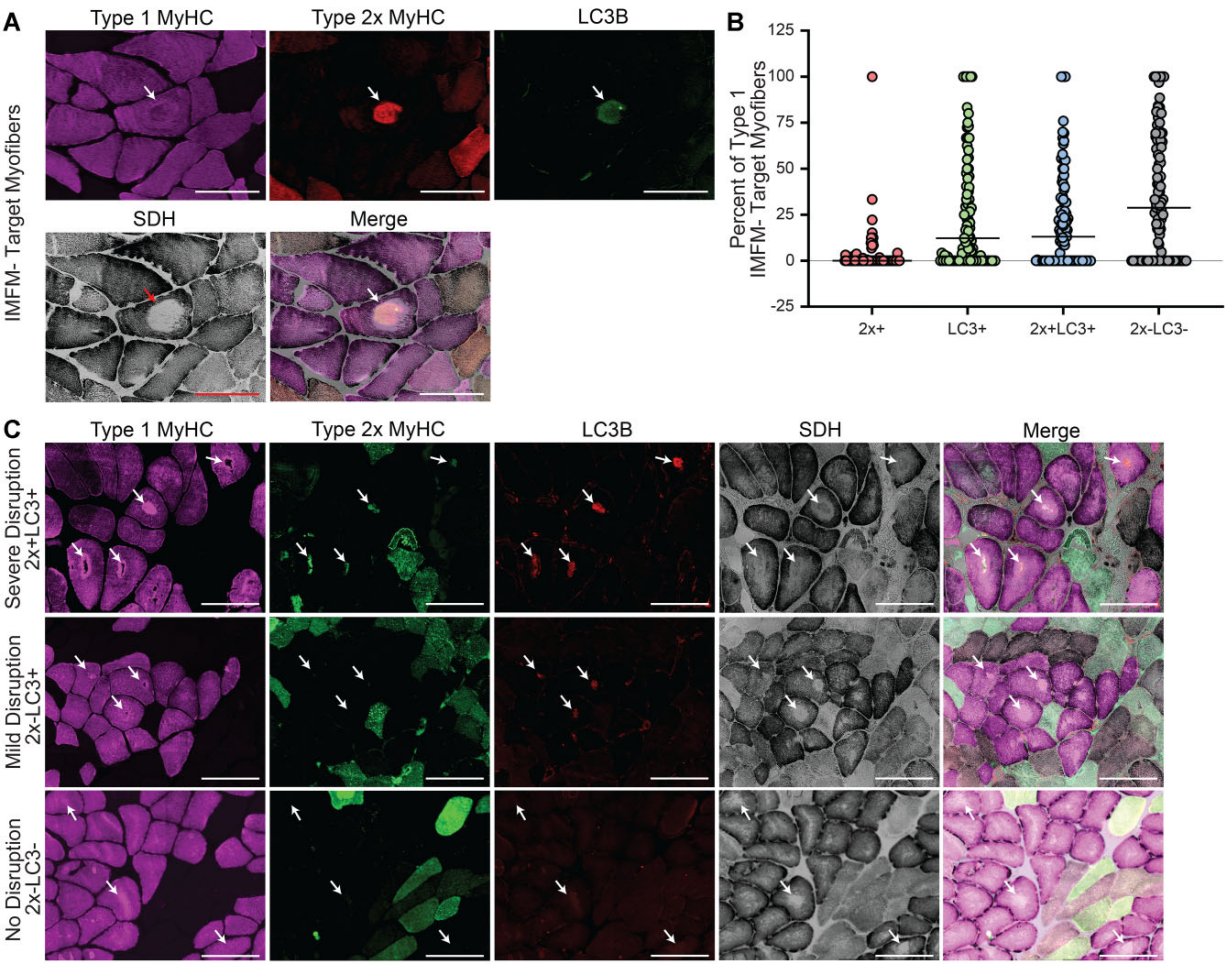
Remnants of 2x MyHC and the autophagy marker LC3B within some Type 1 IMFM- target myofibers. A) Representative ROIs depicting an IMFM- target myofiber with mild disruption of Type 1 MyHC along with 2x MyHC and LC3B within areas devoid of mitochondria (SDH)(arrow). ROIs taken from images acquired at 200x, scale bar = 200 µm. B) Dot plot quantification of Type 1 IMFM- target myofiber characteristics across all samples with IMFM- target myofibers (*n* = 99). Data expressed as median. C) Representative images depicting levels of myosin disruption and presence of 2x MyHC and/or LC3 within IMFM- target myofibers. As Type 1 myosin disruption subsides, 2x MyHC and LC3 disappear, suggesting IMFM- target myofibers may represent a myofiber transition from Type 2 to Type 1 with remnants of damaged 2x being cleared via autophagy while Type 1 MyHC and mitochondria are remodeled.

A significant number of IMFM- areas within type 1 myofibers contained 2x MyHC, often colocalized with LC3, a marker of autophagy (Figure 4A, white arrow). IMFM- myofiber areas were characterized by the presence of 2x MyHC alone (2x+), LC3 alone (LC3+), 2x MyHC colocalized with LC3 (2x + LC3+), or neither 2x MyHC nor LC3 (2x-LC3-) (Figure 4B). On average, 35.4% of IMFM- myofiber areas contained neither 2x MyHC nor LC3 (2x-LC3-). Additionally, 21.3% of IMFM- areas were 2x + LC3 + and 25.4% of IMFM- areas contained only LC3 (2x-LC3+). IMFM- areas containing only 2x MyHC (2x+) were least abundant comprising 2.9% of type 1 IMFM- target myofibers. Within some type 1 myofiber IMFM- areas, type 1 MyHC was either absent (Figure 4C, top) or non-uniformly distributed (Figure 4C, middle). An absence of type 1 MyHC appeared in IMFM- target myofibers containing 2x + LC3 + areas (Figure 4C, top), whereas LC3 + IMFM- areas displayed non-uniform type 1 MyHC (Figure 4C, middle). IMFM- areas lacking both 2x MyHC and LC3 (2x-LC3-) contained intact type 1 MyHC (Figure 4C). Together these findings suggest that some IMFM- areas in type 1 myofibers may contain remnants of a 2a/x myofiber undergoing 2x MyHC autophagy, marked by the presence of LC3.

### Expression of NCAM Within Myofibers of Muscle From Individuals With PAD

**Figure 5.**
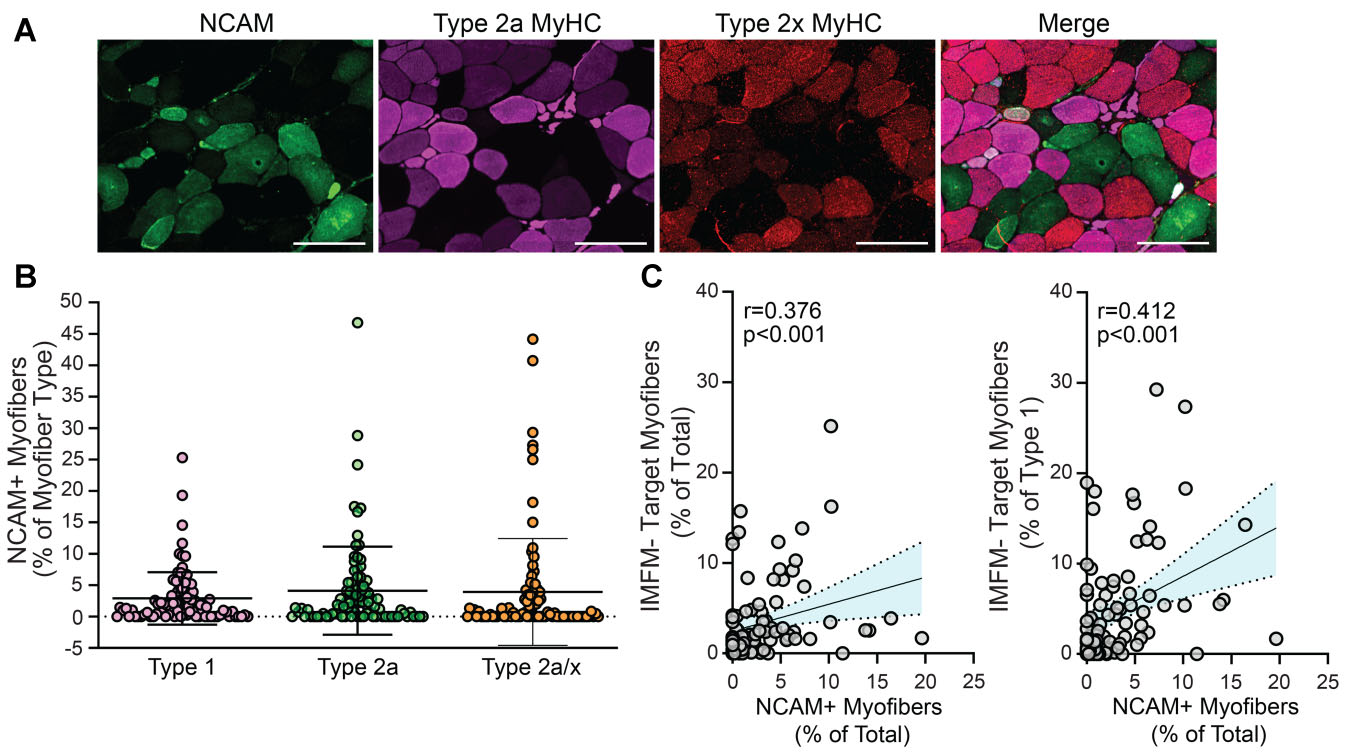
Expression of NCAM in gastrocnemius muscle from individuals with PAD. A) Representative ROIs depicting NCAM+ myofibers along with myofiber type: 2a MyHC and 2a/x MyHC. ROIs taken from images acquired at 200x, scale bar = 200 µm. B) Dot plot quantification of NCAM expression within each myofiber type. Data expressed as mean and standard deviation. C) Representative dot plot showing associations of total NCAM+ myofibers with (Left) the percentage total IMFM- target myofibers and (Right) the percentage of Type 1 IMFM- target myofibers. Associations determined using Spearman Rho, *r* = correlation coefficient, *n* = 90; error bars represent the 95% confidence interval of the linear regression.

NCAM expression has been associated with denervation, disuse, and reinnervation and NCAM+ myofibers are rare or undetectable in adult, non-diseased human muscle.^50-52^ In muscles from individuals with PAD, 80.0% of the samples had at least one NCAM+ myofiber, and overall, 3.2% of total myofibers in a muscle were NCAM+ (Figure 5A). To determine if NCAM was observed more commonly in a specific myofiber type, NCAM expression was analyzed in conjunction with MyHC expression. Expression of NCAM was observed in an average of 4.1% of Type 2a myofibers (range: 0.0%-46.8%) (Figure 5B), 3.9% of 2a/x myofibers (range: 0.0%-44.2%) and 2.9% of type 1 myofibers (range: 0.0%-25.3%) (Figure 5B). Higher proportions of NCAM+ myofibers were associated with higher proportions of total IMFM- target myofibers (0.38, *P* < 0.001) and type1 IMFM- target myofibers (*r* = 0.41, *P* < 0.001) (Figure 5C). Total NCAM+ myofibers were positively associated with both 2a/x MFD (*r* = 0.36, *P* = 0.001) and mean MFD (*r* = 0.30, *P* = 0.004) (Figure S1B). These data show significant NCAM expression in PAD muscle which was associated with larger myofiber size and the presence of IMFM- target myofibers, consistent with classic descriptions of target myofibers demarcating denervation-reinnervation events.^46,53,54^

## Discussion

Among 113 people with PAD, there were substantial interindividual differences in myofiber type distribution within the gastrocnemius muscle. More severe PAD (lower ABI) was associated with a higher proportion of type 1 myofibers and a lower proportion of 2a/x myofibers. Smoking may contribute to myofiber type changes with increasing disease given the diminished association between the proportion of type 1 myofibers and ABI when adjusting for smoking status. However, smoking has also been associated with lower ABI,^55,56^ highlighting the need for future exploration to tease apart contributions between smoking, ABI, and myofiber type composition. Abnormal myofibers lacking intermyofibrillar mitochondria (IMFM- target myofibers) were observed in 76.1% of muscle samples from individuals with PAD and were predominantly type 1. PAD muscles with a higher abundance of IMFM- target myofibers contained higher proportions of type 1 myofibers and lower proportions of type 2a/x myofibers. Type 1 IMFM- myofiber areas contained 2x MyHC, the autophagy marker LC3, both, or neither. These findings suggest that IMFM- target myofibers and contents within IMFM- areas may indicate adaptive remodeling of myofiber type from type 2a/x to type 1 in response to more severe PAD (lower ABI). These findings may provide the first visualization of a myofiber type transition. However, as our data are derived from a single time point, we cannot directly confirm a dynamic myofiber type transition. Longitudinal studies, such as those involving repeat biopsies are required to validate this hypothesis. Further, our inferences regarding ischemia-related muscle changes are based on ABI as a macrovascular surrogate; however, ABI does not directly reflect microvascular perfusion to myofibers which may influence myofiber adaptations independent of ABI values.^57^ Future studies incorporating microvascular assessments would provide deeper insights into the contribution of tissue-level perfusion to myofiber adaptations in PAD.

Transmission electron microscopy demonstrated loss of mitochondria and sarcomere architecture in the IMFM- myofiber areas, confirming the presence of remodeling within the myofiber. The proportion NCAM+ myofibers was positively associated with the abundance of IMFM- target myofibers, suggesting that denervation and the appearance of IMFM- target myofibers occur together. While NCAM provides supportive evidence of denervation, our interpretation is tempered by known caveats.^44^ Future studies could enhance rigor with integrated criteria such as myofiber shape.^45^ Of note, our observation of IMFM- areas predominantly within type 1 myofibers is consistent with previously described target or targetoid myofibers, classically associated with denervation-reinnervation events.^46-49,53,54^ A recent study by Krause et al. explored protein expression within selected target myofibers, reporting proteins related to myofibrillogenesis as the most overrepresented proteins within target myofibers.^46^ The authors suggest target myofibers represent an ongoing process of sarcomere assembly/remodeling in response to myofiber reinnervation, supporting our hypothesis that IMFM- target myofibers indicate myofiber type transitions within PAD muscle.

Quantification of MFD revealed higher proportions of type 1 myofibers were associated with larger overall myofibers while higher proportions of type 2a myofibers were associated with overall smaller myofibers, consistent with prior studies reporting type 2 myofiber atrophy with PAD.^18,58,59^ This study is cross-sectional, and no causal inferences can be made. However, these findings suggest that as disease severity increases (lower ABI) and the impairment of blood flow becomes more severe, oxidative damage-resistant type 1 myofibers become more abundant, consistent with prior studies reporting lower type 2 and higher type 1 myofibers with more severe disease.^18,19^ It is possible that oxidatively damaged 2a/x myofibers are reinnervated by slow motor neurons and remnants of 2x MyHC are cleared through autophagy, then gradually replaced by type 1 MyHC. During this transition, the myofiber remodels, leading to the appearance of IMFM- target myofibers and including expansion of the mitochondrial network, with the intermyofibrillar mitochondria in the center of the myofiber last to be populated (indicated by the IMFM- areas). Further study assessing longitudinal muscle samples and utilizing pre-clinical models are needed to evaluate this hypothesis.

PAD is characterized by many underlying changes shared with the aging process, including oxidative damage, mitochondrial dysfunction, and inflammation.^60-62^ In individuals without PAD, increasing age is accompanied by a decrease in type 2 myofiber number and size, associated with myofiber denervation.^63,64^ A small study of PAD patients with severe claudication (Rutherford category 3) reported significantly greater NCAM+ myofibers in PAD muscles compared to non-PAD.^22^ Findings reported here document NCAM expression within 80% of muscle from PAD participants, averaging >3% of total myofibers, demonstrating a higher prevalence of myofiber denervation in people with PAD than what has been reported in older people without PAD.^51^ Aging has been associated with increases in hybrid myofibers co-expressing multiple MyHC isoforms^65^ and muscle specific changes in myofiber type composition. For example, the human lateral pterygoid muscle of the masseter exhibits an increase in fast MyHC isoforms with aging.^66^ Conversely, the human vastus lateralis muscle has been reported to shift toward higher proportions of type 1 myofibers with aging.^63,67^ During aging in the vastus lateralis, denervated type 2 myofibers can be rescued by reinnervation with a slow motor neuron and conversion to type 1, resulting in type 1 myofiber grouping.^63,67,68^ Prior studies have documented type 1 myofiber grouping in the gastrocnemius muscle from individuals with PAD.^17,26^ Furthermore, evidence from rodent models suggests type 2 myofibers are preferentially damaged and lost with ischemia-reperfusion injury.^69-72^ Analysis of oxidative damage in PAD muscle revealed greater damage in type 2 myofibers, which was associated with reduced myofiber size.^59,72^ Type 2 myofiber atrophy in PAD muscle has also been associated with increases in angular myofibers, consistent with denervation.^18^ In PAD muscle, lower numbers of type 2 myofibers relative to type 1 and more angular myofibers were reported in limbs with lower ABIs compared with contralateral limbs.^73^ Additionally, in vitro studies of C_2_C_12_ muscle cells have shown induction of slow-oxidative type 1 MyHC under hypoxic conditions.^74,75^ Together, these data support the possibility of adaptive myofiber type remodeling under conditions inducing selective type 2 myofiber damage and low oxygen, both present with PAD.

Recent studies have provided molecular comparisons between myofiber types, showing that type 1 myofibers are more resistant to oxidative damage. Hence, under conditions with high oxidative stress, such as PAD, transitions toward type 1 myofibers may be protective against myofiber oxidative damage. First, type 1 myofibers contain mitochondria less prone to the production of reactive oxygen species (ROS) compared to type 2 myofibers.^76,77^ Type 1 myofibers contain greater antioxidant enzyme activity, including catalase, superoxide dismutase, and glutathione.^78^ Thus, type 1 myofibers have a greater capacity to reduce oxygen radicals and mitigate oxidative damage compared with type 2 myofibers. In permeabilized myofiber bundles from rats, the soleus muscle (comprised of 50% type 1 myofibers) exhibited greater scavenging of hydrogen peroxide compared with the EDL muscle (containing > 95% type 2 myofibers).^79^ Proteomic analysis of single muscle fibers from mouse soleus and EDL has further highlighted myofiber type-specific mitochondrial specialization.^77,80^ In one such study, Murgia et al. report higher levels (>30 fold) of the enzymes IDH2 and NNT in type 1 myofibers, both involved in defense against ROS.^77^ Murgia also showed type 1 myofibers have a fivefold higher expression of the repair protein Mitsugumin-53/Trim72, which functions to reseal injured membranes and restore calcium homeostasis.^77^ Together, these findings suggest type 1 myofibers may contain specialized mitochondria, pools of antioxidants, and protein profiles contributing to superior resistance to oxidative damage which may be beneficial in those with PAD.

Our findings suggest heterogeneity in myofiber type composition in PAD may be influenced by disease severity, but these results warrant cautious interpretation due to potential confounding variables such as race, sex, and physical activity. Emerging evidence highlights sex- and race-specific influences on skeletal muscle in PAD, though direct histological or metabolic analyses remain limited. Data may indicate a higher incidence of PAD in women compared to men, and incidence may be even greater in Black and Hispanic women compared to White women.^81-83^ Women with PAD exhibit heightened oxidative stress and impaired skeletal muscle microcirculation, with shorter times to minimum calf muscle hemoglobin oxygen saturation during exercise and elevated reactive oxygen species production.^84^ African American women had greater levels of endothelial ROS production, suggesting this demographic may be particularly susceptible to atherosclerotic progression.^84^ This aligns with greater functional decline in women, including reduced walking distances and higher amputation risks, particularly among Black women with PAD.^81,83^ Although transcriptional profiles of muscle mitochondrial genes appear similar across races in advanced PAD (critical limb ischemia, CLI), other gene signatures differed significantly between Black and White CLI patients.^85^ Further, Black patients experience faster declines in muscle-related mobility, often mediated by socioeconomic factors like income and physical activity levels.^83,86^ Physical activity may promote positive adaptations to PAD,^87^ including improved muscle oxidative metabolism,^73,88^ improved endothelial function,^89,90^ and myofiber denervation-reinnervation.^73^ All these pathways could enhance muscle function and mitigate ischemic damage, though individual responses may vary based on PAD severity, exercise adherence, and comorbidities. While our study did not assess physical activity, the potential influence of physical activity on myofiber composition is acknowledged. The interplay of race, sex, and physical activity in shaping skeletal muscle pathology in PAD remains underexplored, necessitating future research to elucidate independent and combined effects on muscle function and disease progression.

## Study Limitations

First, these data were cross-sectional. No causal inferences can be made. Second, type 2 myofibers contain fewer mitochondria compared to type 1 and the weakest labeling using SDH activity assays. Therefore, IMFM- target myofibers may be easier to identify within type 1 myofibers given the darker labeling. Third, findings of MyHC disruption in conjunction with 2x + LC3 + or LC3 + IMFM- myofibers are qualitative. Fourth, our study is exploratory in nature and correction for multiple testing was not employed to avoid obscuring potentially important associations. Therefore, results should be interpreted with caution as correlations reveal only modest effect sizes.^91^ Thus, future studies employing larger sample sizes are necessary to fully elucidate the biological relevance of the findings we present here. Further, most of the participants in the clinical trials did not have muscle biopsies. Results may not be generalizable to participants without muscle biopsies. The potential contribution of smoking to our findings and the high prevalence of smoking within the PAD patients included in our analyses (50.4%) may limit the generalizability of our findings to non-smoking PAD patients. Our sample size limits the ability to explore potential effects of several key confounding variables (race, sex, physical activity, diabetes, statin use, and others) which likely influence ABI.

Another limitation of this study is the reliance on ABI for perfusion estimates, which does not fully represent the microcirculatory environment feeding myofibers. We cannot rule out the impact of changes in the microcirculatory environment on the variability in myofiber type observed, underscoring the need for multimodal assessment in PAD research. We acknowledge that resting ABI has limited sensitivity and accuracy to fully capture the ischemic burden within the gastrocnemius muscle, particularly under dynamic conditions.^57^ Future studies should include additional diagnostic approaches such as exercise ABI, toe brachial index, doppler waveform, and diagnostic imaging. Finally, functional assessments were not presented in this study as these measurements were beyond the scope of our hypothesis. Thus, our findings should be interpreted with the noted limitations in mind and future studies should be aimed toward better understanding of factors influencing skeletal muscle changes with PAD.

## Conclusions

Large interindividual differences were observed in myofiber type distribution in gastrocnemius muscle among individuals with PAD. Myofibers lacking intermyofibrillar mitochondria (IMFM-) were present in >76% of the samples analyzed and were associated with higher proportions of type 1 myofibers, lower proportions of type 2a/x myofibers, larger myofibers, and more NCAM+ myofibers. IMFM- target myofibers may indicate compensatory myofiber type changes in response to chronic ischemia-reperfusion injury in PAD whereby type 2a/x myofibers transition to more oxidative damage-resistant type 1 myofibers.

## Supplementary Material

zqaf047_Supplemental_Files

## Data Availability

The data underlying this article will be shared on reasonable request to the corresponding author.
