# Supplementary material for: Gastrocnemius Myofiber Type and Mitochondrial Alterations Associated With Peripheral Artery Disease Severity
Source: Function (Oxf). 2025 Oct 6;6(6):zqaf047. doi: 10.1093/function/zqaf047 (PMC12581898; doi:10.1093/function/zqaf047)
Supplement: zqaf047_Supplemental_Files [file zqaf047_supplemental_files.zip › STable 1. Inclusion Exclusion criterion table.docx]

**Supplemental Table 1. Clinical trial inclusion and exclusion criteria.**

| Trial name | Inclusion | Exclusion |
| --- | --- | --- |
| PROPEL | - Baseline ABI ≤0.90 in either leg - Baseline ABI >0.90 with hospital-affiliated vascular lab report/lower extremity angiogram demonstrating PAD - Baseline ABI > 0.90 to 1.00 or normal ABI with prior lower extremity revascularization if ABI decreased by 20% after a heel-rise test (50 heel rises at a rate of 1/sec) | - Below- or above knee amputation - Wheelchair confinement - Use of walking aid other than a cane - Inability or unwillingness to attend exercise sessions - Foot ulcer or critical limb ischemia - Significant visual or hearing impairment - Failure to complete run-in (1 weekly health education session and 1 treadmill exercise session over a 3-week period) - Major surgery or revascularization during the previous 3 months or planned during the next 6 months - Current, or within the past 3 months, participation in a clinical trial or cardiac rehabilitation - Parkinson disease - Require oxygen during activity - Participants for whom exercise may be unsafe – greater than class II New York Heart Association heart failure or angina, increase in angina pectoris during the prior 6 months, or abnormal baseline stress test findings - Already exercising at a level similar to the interventions - Treated for cancer in the past 2 years unless the cancer was early stage and prognosis was excellent - A Mini-Mental Status Examination score less than 23 |
| COCOA-PAD pilot | - Age >60 years - Baseline ABI ≤0.90 in either leg - Baseline ABI >0.90 with hospital-affiliated vascular lab report/lower extremity angiogram demonstrating PAD - The absence of classical symptoms of intermittent claudication was not an exclusion criterion because most people with PAD do not have IC symptoms | - People unwilling to give up major dietary sources of chocolate - Allergy to chocolate or unable to consume products manufactured on equipment processing nuts, egg, wheat, soy, or milk - Major leg amputation - Critical limb ischemia - Wheel-chair confinement or walking aid use - Walking impairment for reasons other than PAD - Significant visual or hearing impairment - People on dialysis - Those requiring oxygen - Major cardiovascular event - Revascularization - Major surgery in the past 3 months - Those planning revascularization or major surgery in the next 6 months - Treated for cancer in the past 2 years unless prognosis was excellent - A baseline 6-minute walk <500 or >1600 feet - A Mini-Mental Status Examination score less than 23 |
| LITE | - Baseline ABI ≤0.90 in either leg - Baseline ABI >0.90 with hospital-affiliated vascular lab report/lower extremity angiogram demonstrating PAD - Baseline ABI >0.90 to 1.00 or normal ABI with prior lower extremity revascularization if ABI decreased by 20% after a heel-rise test (50 heel rises at a rate of 1/sec) - Ischemic leg symptoms during walking no consistent with classic claudication symptoms, such as ischemic leg symptoms affecting the buttocks or thighs but not the calves | - Major amputation - Wheelchair confinement - Use of a walking aid other than a cane - Having a walking limitation for a reason other than PAD - Foot ulcer or critical limb ischemia - Significant visual or hearing impairment - A Mini-Mental Status Examination score less than 23 - Major surgery planned to occur within the next 12 months - Having undergone lower-extremity revascularization or orthopedic surgery during the previous 3 months - Major medical illness - Participants for whom exercise may be unsafe - Already exercising at a level similar to the targeted exercise intervention - Unable to walk sufficiently slowly to avoid ischemic leg symptoms - Those without ischemic leg symptoms during walking |
| TELEX | - Baseline ABI ≤0.90 in either leg - Baseline ABI >0.90 and: - hospital-affiliated vascular lab report showed an ABI <0.90 - toe-brachial index of <0.70 and ischemic leg symptoms - toe-brachial index <0.60 without ischemic leg symptoms - angiogram showing at least 70% stenosis in an artery supplying the legs | - Individuals taking and ACE inhibitor or ARB - Systolic blood pressure <100 mm Hg and diastolic blood pressure <50 mm Hg - Potassium level greater than 5.0 mmol/L - Major amputation - Wheelchair confinement - Use of a walking aid other than a cane - Having a walking limitation for a reason other than PAD - Foot ulcer or critical limb ischemia - Significant visual or hearing impairment - A Mini-Mental Status Examination score less than 23 - Having undergone lower-extremity or coronary revascularization or major surgery during the previous 3 months - Planned revascularization or major surgery within the next 6 months - Major medical illness - Participants for whom exercise may be unsafe - Already exercising at a level similar to the targeted exercise intervention - Those who did not complete at least 10 days of the 14-day run-in period - Those with any of the following at the end of the run-in period: - new lightheadedness - potassium level ≥5.5 mmol/L - a decrease in estimated glomerular filtration rate by 30% or greater - a systolic blood pressure <100 mm Hg and a diastolic blood pressure <50 mm Hg |
| WALCS III | - for participants with PAD: lowest leg ABI ≤0.999 - for participants in the control group: lowest ABI between 1.00 to 1.30 | - Foot amputation, leg amputation or gangrene - Recent liver transplant - Requires oxygen on ambulation - End stage renal disease - Confined to a wheelchair - Lives in a nursing home - Low life expectancy - A Mini-Mental Status Examination score less than 23 - Communication difficulty due to language barriers - Inability to tolerate MRI testing for any reason - 6-minute walk performance limited primarily by pulmonary disease, legal blindness, or severe lower extremity arthritis |
| BRAVO | - Baseline ABI ≤0.90 in either leg - Baseline ABI ≥0.90 with documentation of PAD from an accredited vascular laboratory - Lower-extremity revascularization for PAD | - ABI >0.90 and no documented evidence of PAD - A Mini-Mental Status Examination score less than 23 or history of cognitive impairment - Refusal to have regular blood draws or inability to obtain a blood sample at baseline - Coronary or cerebrovascular event during the previous 6 months - History of inflammatory arthritis (rheumatoid arthritis, lupus erythematosus, or polymyalgia rheumatica, gout within the last 3 months) - Not from an acceptable recruitment source - Treatment for cancer other than non-melanoma skin cancer during the previous 2 years (those treated for non-invasive breast cancer or prostate cancer during the pervious year were potentially eligible) - Unintentional weight loss of more than 7.5 lb in the last 6 months - Communication difficulty due to language barriers - Residence more than 40 Mi away from the medical center and unwillingness to travel to the medical center for every two-month blood collection - Unable to return for follow-up testing for ≥a consecutive 6-month period in the next 2 years - Heart transplant surgery - Major surgery within the past 3 months - Currently enrolled in a clinical research trial or another study with the same principal investigator (MMM) |
| GOALS | - Baseline ABI ≤0.90 in either leg - Baseline ABI >0.90 with documentation of PAD from an accredited vascular laboratory - Medical record documentation of lower extremity revascularization | - Below- or above-knee amputation - Wheelchair confinement - Inability to walk 50 ft without stopping - Use of a walking aid other than a cane - Inability to attend weekly sessions - Walking impairment for a reason other than PAD - Foot ulcer or critical limb ischemia - Significant visual or hearing impairment - Non-completion of the study run-in (attendance at 2 weekly health education sessions over a 3-week period) - Major surgery or lower extremity revascularization during the previous 3 months or planned surgery during the next 12 months - Major medical illness, including cancer treatment during the prior 12 months - Current participation in another clinical trial or in another exercise trial within the past 3 months - Completion of cardiac rehabilitation during the past 3 months - Parkinson disease - Requirement of oxygen with activity or exercise - Participants for whom exercise may be unsafe including having more than a class II New York Heart Association heart failure or angina - An increase in angina pectoris during the prior 6 months - Abnormal baseline exercise stress test - An exercise level similar to that targeted in the intervention at the time of recruitment - A Mini-Mental Status Examination score less than 23 |
| RESTORE | - Age ≥65 years - Presence of PAD: baseline ABI <0.90, medical record-documented lower extremity revascularization, or noninvasive vascular laboratory rest results consistent with PAD | - Below- or above-knee amputation - Wheelchair confinement - Use of a walking aid - Walking impairment for a reason other than PAD - Significant visual or hearing impairment - Required dialysis - Lung disease requiring oxygen - Substantial liver disease - Major cardiovascular event, major surgery, or endovascular revascularization during the previous 3 months - Planned lower extremity revascularization or major surgery in the next 6 months - A Mini-Mental Status Examination score less than 23 - Already participating in another clinical trial - Treated for cancer in the past 2 years (unless their prognosis was excellent) - Currently taking or allergic to resveratrol - Had a baseline 6-minute walk test of <152.4 m or >487.7 m - Did not take at least 80% of daily placebo pills during a 2-week study run-in |
| PERMET | - Baseline ABI ≤0.90 in either leg - Baseline ABI >0.90 with hospital-affiliated vascular lab report or angiographic evidence of PAD who have ischemic symptoms during the 6-minute walk and/or treadmill exercise stress test - Baseline ABI >0.90 to 1.00 and experience a 20% or higher drop in ABI after a heel-rise exercise - A history of lower extremity revascularization who do not meet the criterion above, have an ABI >0.90, and experience a 20% or higher drop in ABI after a heel-rise exercise | - Below- or above-knee amputation - Critical limb ischemia - Wheelchair bound or requiring a walker to ambulate - Walking impairment for a reason other than PAD - Foot ulcer on bottom or foot - Diabetes mellitus defined as one or more of: - Patient report of physician diagnosed diabetes mellitus - Use of one or more diabetes medications - Two baseline hemoglobin A1C values of >6.5 - Two fasting glucose values >126 mg/dl - Chronic kidney disease defined as GFR ≤45 - Chronic liver disease defined as two or more hepatic function tests ≥2 times the upper limit of normal - Failure to successfully complete the 2-week study run-in, defined as unable to tolerate metformin and/or failing to take the medication daily for 10 or more days in the two-week period - Planned lower extremity revascularization, orthopedic surgery, or other major surgery during the next six months - Lower extremity revascularization, orthopedic surgery, cardiovascular event, coronary revascularization or other major surgery in the previous 3 months - Major medical illness including renal disease requiring dialysis, lung disease requiring oxygen, Parkinson’s disease, a life-threatening illness with life expectancy less than 6 months, or cancer requiring treatment in the previous 2 years (NOTE: potential participants may still qualify if they have had treatment for an early stage cancer in the past two years and the prognosis is excellent. Participants who only use oxygen at night may still qualify.) - Mini-Mental Status Examination score less than 23 or dementia. - Participation in or completion of a clinical trial in the previous 3 months. - Currently taking metformin or has taken metformin in the past 6 months - Increase in angina or angina at rest - Non-English speaking - Visual impairment that limits walking ability - Thos for whom the investigator determines the trial to be unsafe or not a good fit |
| HI-PAD | - Age ≥55 years - Baseline ABI ≤0.90 in either leg   Baseline ABI >0.90 with vascular laboratory or angiographic evidence of PAD in either leg and ischemic calf symptoms, defined as exertion-induced calf symptoms during the 6-minute walk test, baseline exercise stress test, or daily walking activities | - Chronic limb-threatening ischemia - Lower extremity ulcers whose walking was limited by symptoms other than PAD - Required any assistive device for ambulation - Planned lower extremity revascularization, major orthopedic or other major surgery in the next 6 months - Lower extremity or coronary revascularization, orthopedic surgery, a cardiovascular event, or major surgery in the previous 3 months - Major medical illness including: - kidney disease requiring dialysis - lung disease requiring oxygen - Parkinson disease - Life expectancy of less than 6 months - A Mini-Mental Status Examination score less than 23 - To avoid potential adverse events during receipt of VM202, potential participants with the following were excluded: - Proliferative retinopathy (people with diabetes were assessed by a study investigator) - Diagnosed with cancer in the past 5 years (except for cancers with an excellent prognosis) - Not current with cancer screening recommendations - Tested positive for and had a measurable viral load for HIV, hepatitis B, or hepatitis C - Significantly abnormal hemoglobin, white blood count, or liver function testing - Recent increase in chest pain - Premenopausal women - Baseline 6-minute walk <595 feet or >1520 feet - Participation in a supervised exercise therapy or plans to begin a supervised exercise therapy during the clinical trial |
