# Supplementary material for: Gastrocnemius Myofiber Type and Mitochondrial Alterations Associated With Peripheral Artery Disease Severity
Source: Function (Oxf). 2025 Oct 6;6(6):zqaf047. doi: 10.1093/function/zqaf047 (PMC12581898; doi:10.1093/function/zqaf047)
Supplement: zqaf047_Supplemental_Files [file zqaf047_supplemental_files.zip › STable 2. Major Resources Table.docx]

**Supplemental Table 2: Major Resources Tables**

| **Antibody target/reagent** | **Vendor or Source** | **Catalog #** | **Dilution/working concentration** | **Antibody Registry ID** |
| --- | --- | --- | --- | --- |
| **Myofiber type distribution and minimum ferret diameter** | | | | |
| Anti-mouse IgG2b Type 1 MyHC | Developmental Studies Hybridoma Bank (DSHB) | BA.D5-c | 1:100 | RRID: AB_2235587 |
| Anti-mouse IgG1 Type 2a MyHC |  | SC.71-s | 1:50 | RRID: AB_2147165 |
| Anti-mouse M Type 2x MyHC |  | 6H.1-s | Use as diluent | RRID: AB_1157897 |
| Laminin | Millipore Sigma | L9393 | 6.5 µg/mL | RRID: AB_477163 |
| Goat anti-mouse IgG2b Alexa Fluor 647 | Thermo Fisher | A21242 | 1:250 | RRID: AB_2535811 |
| Goat anti-mouse IgG1 Alexa Fluor 488 |  | A21121 | 1:250 | RRID: AB_2535764 |
| Goat anti-mouse IgM Alexa Fluor 555 |  | A21426 | 1:250 | RRID: AB_2535847 |
| Biotinylated goat anti-rabbit | Jackson ImmunoResearch | 111-065-144 | 1:1000 | RRID:AB_2337965 |
| Streptavidin AMCA | Vector Laboratories | SA-5008-1 | 1:150 | RRID:AB_2336103 |
| **Succinate Dehydrogenase (SDH)** | | | | |
| **0.2 M PBS, pH 7.4** | | | | |
| NaH2PO4.H2O | Fisher Scientific  Fisher | S397 | 0.54 g | |
| Na2HPO4.7H2O |  | S373 | 4.29 g | |
| Distilled (DI) H2O | N/A | | to 100 mLs | |
| **Solutions** |  | |  | |
| Nitrotetrazolium Blue Chloride (NBT) | Millipore Sigma | N6876 | 30 mg/12 mL DI water | |
| Succinic acid disodium salt |  | 224731 | 0.97 g/18 mL DI water | |
| **SDH Solution** | | | | |
| NBT solution | Recipes above | | 12 mL | |
| Succinic Acid solution |  |  | 18 mL | |
| 0.2 M PBS |  |  | 18 mL | |
| **Bluing/finishing** | | | | |
| Acetone | VWR | BDH1101-1LP | 30% and 60% in DI water | |
| **IMFM- characterization following SDH** | | | | |
| **Blocking** | | | | |
| 2.5% Normal Horse Serum | Vector laboratories | S-2012 | Use as diluent | |
| Tween-20 | Fisher Scientific |  | 0.5% (5 µl/mL) | |
| Anti-mouse IgG2b Type 1 MyHC | Developmental Studies Hybridoma Bank (DSHB) | BA.D5-c | 1:100 | RRID: AB_2235587 |
| Anti-mouse M Type 2x MyHC |  | 6H.1-s | Use as diluent | RRID: AB_1157897 |
| Anti-rabbit LC3B | Novus Biologics | NB100-2220 | 1:100 | RRID:AB_10003146 |
| 4’,6-diamidino-2-phenylindole (DAPI) | Thermo Fisher | D1306 | 0.5 µg/mL | RRID:AB_2629482 |
| Goat anti-mouse IgG2b Alexa Fluor 647 |  | A21242 | 1:250 | RRID: AB_2535811 |
| Goat anti-mouse IgM Alexa Fluor 488 |  | A21042 | 1:250 | RRID:AB_2535711 |
| Biotinylated goat anti-rabbit | Jackson ImmunoResearch | 111-065-144 | 1:1000 | RRID:AB_2337965 |
| Streptavidin Alexa Fluor 594 | Thermo Fisher | S32356 | 1:500 | N/A |
| **Mitochondrial complex staining** | | | | |
| Paraformaldehyde | Fisher Scientific | AAA113130E | 4% in 1x phosphate buffered saline | |
| **50 mM tris buffered saline (TBS)** | | | | |
| Tris Base | Fisher Scientific | BP152-500 | 50 mM (6.05g/L) | |
| Sodium Chloride | Fisher Scientific | BP358-1 | 150 mM (8.76 g/L) | |
| DI water | N/A | | To 1 L | |
| **Blocking** | | | | |
| TBS | Recipe above | |  | |
| Normal goat serum | Vector Laboratories | S1000 | 5 % | |
| Tween-20 |  |  | 0.5% | |
|  |  |  |  |  |
| MTCO1 [1D6E1A8] | Abcam | ab14705 | 1:50 | RRID:AB_2084810 |
| NDUFB8 [20E9DH10C12] |  | ab110242 | 1:100 | RRID:AB_10859122 |
| Biotinylated goat anti-mouse IgG1 | Jackson ImmunoResearch | 115-065-205 | 1.2 µg/mL | RRID:AB_2338571 |
| Goat anti-mouse IgG2a Alexa Fluor 488 | Thermo Fisher | A21131 | 1:250 | RRID:AB_2535771 |
| Streptavidin-horse radish peroxidase |  | S911 | 5 µg/mL (2.5 µg/µl stock) | N/A |
| Superboost TSA Alexa Fluor 594 |  | B40957 | Per manufacturer’s instruction | |
| **Myofiber type specific NCAM** | | | | |
| Anti-mouse IgG1 Type 2a MyHC | Developmental Studies Hybridoma Bank (DSHB) | SC.71-s | 1:50 | RRID: AB_2147165 |
| Anti-mouse M Type 2x MyHC |  | 6H.1-s | Use as diluent | RRID: AB_1157897 |
| Rabbit anti-NCAM | Millipore Sigma | AB5032 | 1:50 | RRID:AB_11213653 |
| Goat anti-mouse IgG2b Alexa Fluor 647 | Thermo Fisher | A21242 | 1:250 | RRID: AB_2535811 |
| Goat anti-mouse IgM Alexa Fluor 555 |  | A21426 | 1:250 | RRID: AB_2535847 |
| Goat anti-rabbit Alexa Fluor 488 |  | A11034 | 1:200 | RRID:AB_2576217 |
