# Supplementary material for: Gastrocnemius Myofiber Type and Mitochondrial Alterations Associated With Peripheral Artery Disease Severity
Source: Function (Oxf). 2025 Oct 6;6(6):zqaf047. doi: 10.1093/function/zqaf047 (PMC12581898; doi:10.1093/function/zqaf047)
Supplement: zqaf047_Supplemental_Files [file zqaf047_supplemental_files.zip › Supplemental Figure 1.docx]

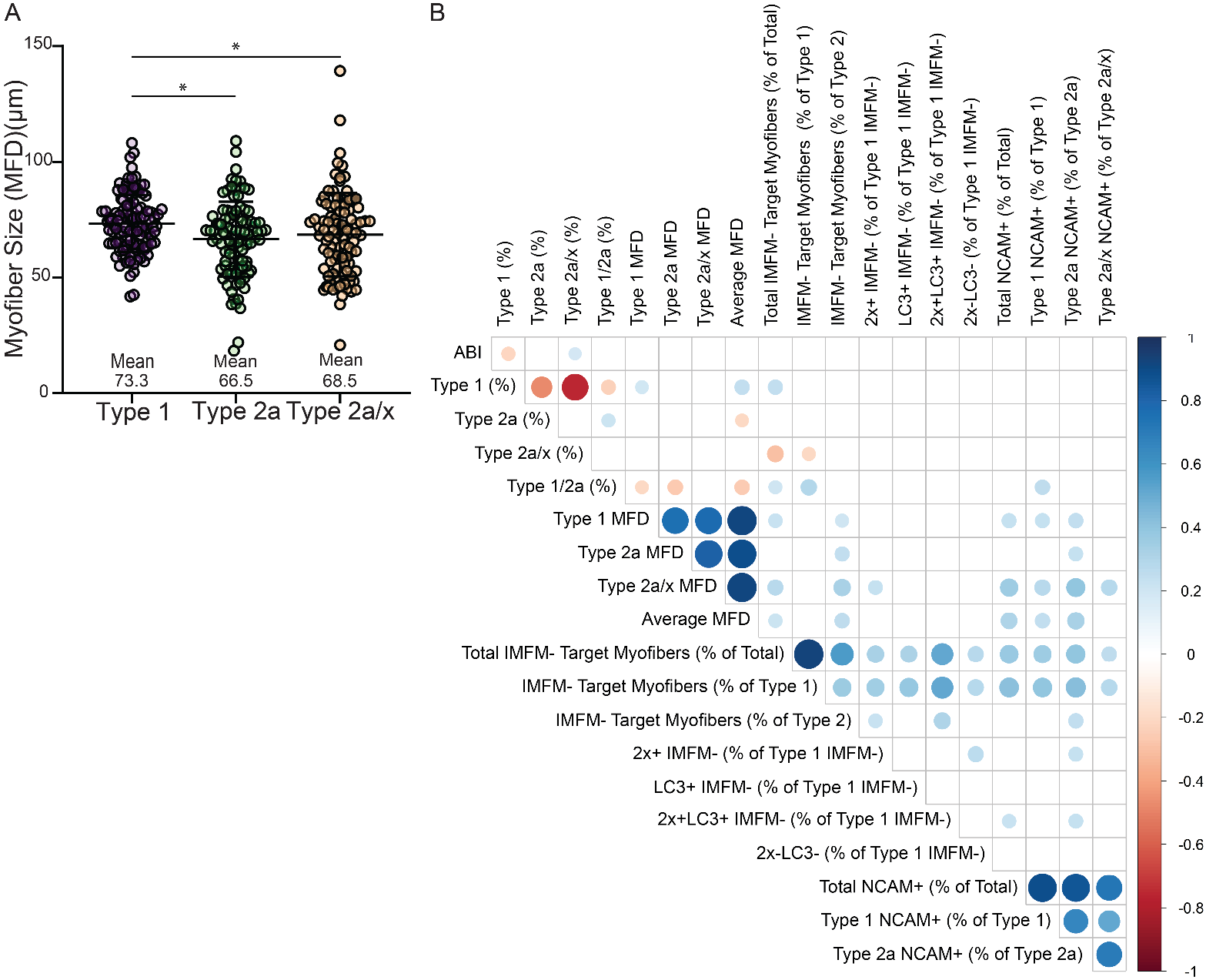


**SFigure 1. Myofiber type size and spearman correlation matrix of muscle features at baseline and 6-month change.** A) Representative dot plot showing myofiber size measured by minimum ferret diameter (MFD) from 113 PAD patient gastrocnemius muscles. Statistical differences assessed using ANOVA with Freidman test and multiple comparisons; *p<0.05. B) Correlation matrix showing associations among all baseline PAD muscle features and ABI. Color intensity and size are related to the correlation coefficients defined in the legend to the right; blue = positive correlation, red = negative correlation.
